# The course of primary progressive aphasia diagnosis: a cross-sectional study

**DOI:** 10.1186/s13195-022-01007-6

**Published:** 2022-05-10

**Authors:** A. Mouton, A. Plonka, R. Fabre, T. M. Tran, P. Robert, J. Macoir, V. Manera, A. Gros

**Affiliations:** 1 Centre Hospitalier Universitaire de Nice, Laboratoire CoBTeK, Service Clinique Gériatrique du Cerveau et du Mouvement, Université Côte d’Azur, Nice, France; 2 Institut NeuroMod, Université Côte d’Azur, Sophia Antipolis, France; 3 Laboratoire STL, UMR 8163, Université de Lille, Lille, France; 4 Faculté de Médecine de Nice, Département d’Orthophonie, Université Côte d’Azur, Nice, France; 5 Department of rehabilitation, Faculty of Medicine, Laval University, Quebec, Canada; 6 CERVO Brain Research Center, Quebec, QC Canada; 7 Laboratoire CoBTeK, Université Côte d’Azur, Nice, France

**Keywords:** Primary progressive aphasia, Alzheimer’s disease, Diagnosis

## Abstract

**Background:**

The primary progressive aphasia (PPA) diagnosis trajectory is debated, as several changes in diagnosis occur during PPA course, due to phenotype evolution from isolated language alterations to global cognitive impairment.

The goal of the present study, based on a French cohort, was to describe the demographics and the evolution of subjects with (PPA) in comparison with Alzheimer’s disease (AD) on a period of 7 years.

**Methods:**

We conducted a repeated cross-sectional study. The study population comprised individuals with PPA and AD diagnosis (*N*=167,191) from 2010 to 2016 in the French National data Bank (BNA). Demographic variables, MMSE scores, diagnosis status at each visit and prescribed treatments were considered.

**Results:**

From 2010 to 2016, 5186 patients were initially diagnosed with PPA, 162,005 with AD. Compared to AD subjects, significant differences were found concerning age (younger at first diagnosis for PPA), gender (more balanced in PPA), education level (higher in PPA) and MMSE score (higher of 1 point in PPA).

Percentage of pending diagnosis, delay between first consultation and first diagnosis and the number of different diagnoses before the diagnosis of interest were significantly higher in PPA group compared to AD group. Pharmacological and non-pharmacological treatments were significatively more recommended following PPA than AD diagnosis.

**Conclusion:**

This study improves the knowledge of PPA epidemiology and has the potential to help adopting appropriate public health service policies. It supports the hypothesis that PPA is diagnosed later than AD. The PPA diagnosis increases the prescription of non-pharmacological treatments, especially speech and language therapy (SLT) that is the main treatment available and most effective when at the initial stage.

**Trial registration:**

ClinicalTrials.gov identifier NCT03687112

**Supplementary Information:**

The online version contains supplementary material available at 10.1186/s13195-022-01007-6.

## Background

Current diagnosis classification identifies three PPA subtypes: the agrammatic subtype (nfavPPA), the semantic subtype (svPPA) and the logopenic subtype (lvPPA).

The age of onset of PPA is usually between 50 and 65 years [1, 2]. PPA ultimately leads to dementia, and the survival duration is estimated between 10 and 15 years [3]. No disease-modifying pharmacological intervention treatment is available so far. However, non-pharmacological interventions, such as speech and language therapy (SLT), have proven to be useful to compensate and maintain functional communication.

Proper PPA diagnosis increases the opportunities of providing early appropriate clinical interventions, implementing coordinated care plans, managing symptoms, improving patient safety, cost savings and postponing institutionalization [4]. The neurodegenerative diagnosis trajectory is still debated, as several changes in diagnosis occur during the course of PPA, due to phenotype evolution from isolated language alterations to global cognitive impairment with associated multiple neuropsychiatric symptoms [5, 6]. Furthermore, lvPPA is considered as an atypical phenotype of Alzheimer’s disease (AD), which further complicates diagnosis [7, 8]. Even if the PPA duration is estimated at about 6 years before dementia onset, language symptoms could represent the only set of signs for as many as 10–14 years.

After a few years of disease progression, deficits in other cognitive domains than language appear, such as episodic memory or executive functions. However, the language dysfunction remains the most salient feature throughout the degeneration process [9, 10]. The diagnosis of PPA is a major challenge in clinical practice as this phenotype is complex and constantly evolving.

Despite PPA has been object of investigation in several studies, given its low prevalence, most of the existing literature deals with small sample sizes, which limits the statistical power and the generalizability of the results. The main objective of this study was to describe the clinical characteristics and the evolution in diagnosis of PPA in comparison with AD, over a period of 7 years, in a large cohort of memory-clinic patients. The secondary objectives were to determine if the diagnosis of PPA is more difficult to establish (more changes in diagnosis before the PPA diagnosis) and more delayed, compared to AD diagnosis, and to compare the two syndromes according to the recommended therapeutic approaches.

## Methods

### Participants

Participants of the present study were recruited from the French National data Bank (BNA) which is part of the French strategy to fight against dementia [11] and records information since the end of 2009. This database was created to provide information about the medical activity of the French memory centres in order to adapt healthcare provision, and generate epidemiologic knowledge on the diseases and the medical practices. The BNA includes a limited set of demographic, diagnostic and clinical information, selected by a national consensus group. The number of collected variables was limited to facilitate and enhance care providers to participate to this national database. Data are collected from 536 memory units in France: 434 memory centres (secondary level), 28 resource and research memory centres (tertiary level) and 74 independent neurologists who expressed the willingness to participate.

Each time a person consults one of these centres, a clinical record is generated and transferred to the database. Therefore, one patient can figure more than once in the BNA, depending on the number of medical acts he/she underwent.

The following variables were considered in the present study: gender, age, living conditions, education (five levels according to the French education system, corresponding to the following categories: no formal education, primary school level [equivalent to 1–5 years of education], secondary school level with 6–9 years of education, secondary school level with 10–12 years of education and university level [over 12 years of education]), type of medical centre, referring modalities, score on the Mini Mental Score Examination (MMSE) [12] date of consultation, medical diagnosis and recommended treatments.

The BNA differentiates 38 diagnostic groups, based on International Classification of Diseases, Tenth Revision, ICD-10. For the AD diagnosis, ICD-10 criteria include insidious and irreversible onset dementia and clinical examination or special investigation that do not suggest any other aetiology of the disorders (metabolic disorder, cerebral haematoma…). Therefore, AD diagnosis was established on clinical, biological and cerebral imaging results. As the BNA is a large databank, reflecting usual clinical practice, on the period studied (2010 to 2016), no metabolic imaging or amyloid proof was required. For treatments, the BNA records the presence of a prescription at the time of the consultation for 6 groups of psychotropic drugs classified as follows, using ATC codes: antidepressant (N06A), anxiolytic (N05B), hypnotic (N05C), antipsychotic (N05A), cholinesterase Inhibitors (ChEIs) (N06DA) and N-Methyl-D-aspartate receptor antagonist (NMDA antagonist) (N06DX01). No data is available on drug generics or brand names, nor on dosage. Psychosocial intervention and rehabilitation are recorded too. More details on this database are described in Le Duff et al. [13].

### Study design and participant selection

Patients were selected in the BNA from January 1, 2010, to December 31, 2016. Patients with at least once a diagnosis of PPA according to the diagnostic criteria including progressive language disorders were included in the PPA group (including all PPA subtypes) [14, 15]. Patients with at least once a diagnosis of AD, but never of PPA were included in the AD group.

**Fig. 1:**
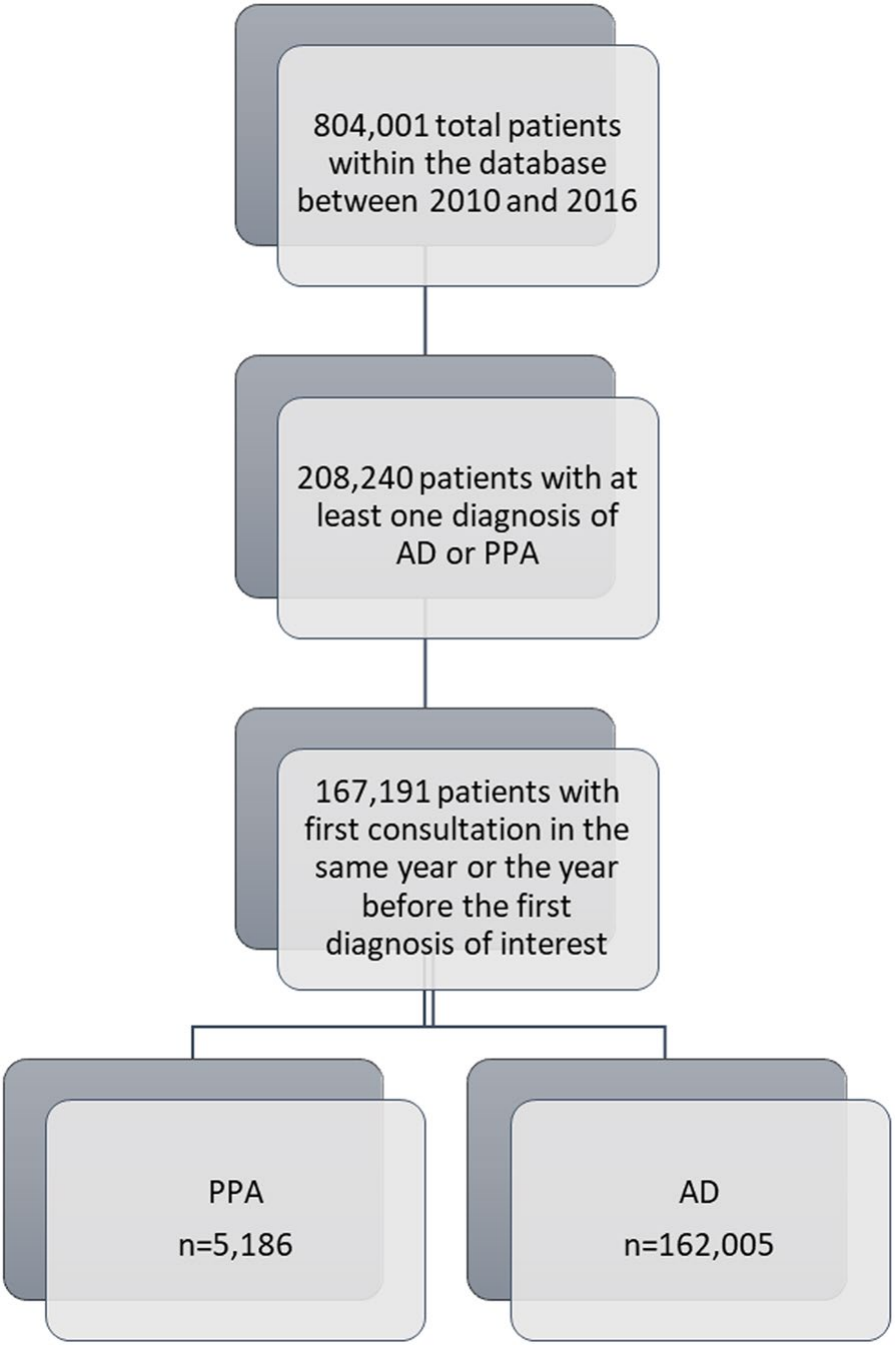
Flowchart: selection of the participants included in the study

Individuals who already had the diagnosis of interest when first registered in the database were included only if their first consultation for memory problems was in the same year or the year before the first visit. This was intended to exclude patients who had a diagnosis established for a long time, and to collect data at the time of the first diagnosis. To describe the whole population included in the study, we selected data at the first diagnosis of interest. Given the importance of cognitive status, only patients with at least one existing MMSE evaluation at less than 1 year before or after the first diagnosis of interest were considered in the analyses (see Fig. 1).

### Statistical analysis

Incident cases were defined as those first diagnosed during the study period. Incidence was calculated by dividing the total number of incident cases by the total number of person-years for the catchment area population over 7 years (data from the French national institute for statistical and economic studies INSEE).

Descriptive analyses were conducted using percent and frequency for qualitative variables and mean with SD for quantitative variables. Variables associated with diagnosis (i.e., PPA, AD) were analysed using Student *t*-test for quantitative variables and chi-squared tests for qualitative variables. The change in treatment and the change in psychosocial interventions were determined using the McNemar test. In all analyses, a *p* value less than 0.05 was considered significant.

In addition, because of the large size of our cohort, we decided to run a second type of analysis: Bayesian analysis. This analysis was also performed as a simple way to deal with significantly labelled differences between large-sized groups. Here we used a burn-in of 1000 iterations (to allow Markov chains to reach stationary distribution) and 4000 useful iterations for estimates. Furthermore, the Bayesian techniques allow acceptance of a null hypothesis (not only rejection), which is not only a comparison with 0 (for example, for a difference). Statistical analyses were done with SAS Enterprise Guide software, version 5.1 (SAS Institute, Cary, NC, USA). Bayesian analyses were done with WinBugs 1.4 software.

## Results

### Clinical characteristics of the 2 groups

The PPA and the AD groups respectively included 5186 and 162,005 patients. The incidence rate of PPA was 1.14 per 100,000 person-years, while the incidence rate of AD was 35.7 per 100,000 persons-years. Demographic characteristics of the two groups are presented in Table 1, and the results of Bayesian analysis are reported in Additional file 1: Table S1. Patients with PPA were significantly younger (mean = 73.7; SD = 9.1 years) than those with AD (mean = 81.4; SD = 8.0 years (*p*<0.001)), and this was observed in all age groups, except for the patients aged 80 years and older for which the opposite pattern was observed. As shown in Table 1, the sex ratio was more balanced in the PPA than in the AD group, and the educational level was higher in the PPA than in the AD group, with a larger proportion of patients with more than 6 years of education (secondary second school level).

**Table 1:** Demographic characteristics

	PPA (*n*=5186)		AD (*n*=162,005)		
	Mean	[SD]	Mean	[SD]	*p*-value
**Age when first diagnosed, years**	73.7	[9.1]	81.4	[8.0]	<.001
**MMSE at ±1 year after diagnosis**	19.5	[7.3]	17.9	[5.9]	<.001
	*n*	(%)	*n*	(%)	*p*-value
**Gender**					<.001
Female	2887	(55.7)	112,751	(69.6)	
Male	2299	(44.3)	49,254	(30.4)	
**Type of centre**					<.001
Memory clinic	2739	(52.8)	120,750	(74.5)	
Regional specialized memory clinic	2309	(44.5)	37,361	(23.1)	
Private neurologist	138	(2.7)	3894	(2.4)	
**Age at first consultation following diagnosis, years**					<.001
< 50	38	(0.7)	462	(0.3)	
[50, 55[	89	(1.7)	567	(0.4)	
[55, 60[	205	(4.0)	1493	(0.9)	
[60, 65]	533	(10.3)	3038	(1.9)	
[65, 70]	807	(15.6)	6694	(4.1)	
[70, 75]	966	(18.6)	14,801	(9.1)	
[75, 80]	1191	(23.0)	31,774	(19.6)	
[80, 85]	923	(17.8)	47,930	(29.6)	
≥ 85	834	(8.4)	55,246	(34.1)	
**Education**					<.001
No education	205	(4.0)	12,523	(7.7)	
Primary	1536	(29.6)	75,615	(46.7)	
Secondary first cycle	1067	(20.6)	26,893	(16.6)	
Secondary second cycle	805	(15.5)	14,715	(9.1)	
Superior	1022	(19.7)	13,222	(8.2)	
Unknown	551	(10.6)	19,037	(11.8)	
**Initially referred by**					<.001
General practitioner	2839	(54.7)	106,157	(65.5)	
Neurologist	1152	(22.2)	9751	(6.0)	
Other specialists	592	(11.4)	17,665	(10.9)	
Direct	233	(4.5)	6974	(4.3)	
Others	370	(7.1)	21,458	(13.3)	
**Community-dwelling**					<.001
No	333	(6.4)	27,240	(16.8)	
Yes	4853	(93.6)	134,765	(83.2)	
**Patient location**					<.001
Within 50 km from the memory clinic	4245	(81.9)	148,844	(91.9)	
Over 50 km from the memory clinic	941	(18.2)	13,161	(8.1)	

Compared to the AD group, the patients of the PPA group were more often referred by neurologists and less by general practitioners. In PPA more often than in AD, the diagnosis was established in a tertiary centre, and patients lived farther from the centre.

In the PPA group, the MMSE score at first diagnosis was significantly different than in AD. Using Bayesian analytical methods, we found that there was a significant difference of 1 point between the two groups, while the difference of 2 points for MMSE was not significant. Patients were more to live in community compared to the patients with AD.

### Evolution in diagnosis

As shown in Additional file 1: Table S2, the delay between the first consultation for cognitive disorders (that could be prior the first record in the BNA) and the first diagnosis visit was significantly longer in the PPA than in the AD group.

The number of different diagnoses before the diagnosis of interest was significantly different in the PPA group than in the AD group. Indeed, the mean number of diagnoses before the diagnosis of interest was 0.54 (SD=0.69) in the PPA group, and 0.45 in the AD group (SD=0.62). The mean time between the first consultation and the first diagnosis was 0.7 years in the PPA group and 0.6 years in the AD group (*p*<0.001) (see Additional file 1: Table S2).

We analysed the diagnoses made before the diagnosis of interest in each group (Table 2). Except “pending diagnosis”, the most frequent diagnosis given before PPA diagnosis were AD (12.6%) then subjective cognitive complaint, followed by non-amnestic mild cognitive impairment. Though before AD diagnosis, except “pending diagnosis”, it was most often amnestic mild cognitive impairment (14%) then cognitive complaint then non-amnestic mild cognitive impairment.

**Table 2:** Diagnoses before and after first diagnosis of interest

	PPA		AD	
	*n*	(%)	*n*	(%)
Diagnosis BEFORE first diagnosis of interest				
Pending diagnosis	3277	(49.6)	83,967	(53.4)
Alzheimer’s disease (AD)	835	(12.6)	0	(0.0)
Memory complaints	492	(7.4)	11,781	(7.5)
MCI	467	(7.1)	10,256	(6.5)
Amnestic MCI	294	(4.4)	21,974	(14.0)
Huntington disease	205	(3.1)	754	(0.5)
Mixed dementia	177	(2.7)	11,348	(7.2)
Anxiety disorder, depressive disorder	177	(2.7)	5540	(3.5)
Frontotemporal lobar degeneration (FTLD)	90	(1.4)	280	(0.2)
Others	596	(9.0)	11,419	(7.3)
Diagnosis AFTER first diagnosis of interest				
PPA	10,760	(72.7)	0	(0.0)
Alzheimer’s disease (AD)	2049	(13.8)	318,769	(90.7)
Frontotemporal lobar degeneration (FTLD)	328	(2.2)	672	(0.2)
Pending diagnosis	323	(2.2)	3761	(1.1)
Huntington disease	229	(1.5)	634	(0.2)
Mixed dementia	223	(1.5)	14,267	(4.1)
MCI	146	(1.0)	1924	(0.5)
Amnestic MCI	89	(0.6)	2546	(0.7)
Others	1107	(7.5)	27,576	(7.8)

The proportion of patients having received no other diagnosis after PPA was identified was lower than after an AD diagnosis was made (see Additional file 1: Table S2).

However, the proportion of patients having received more than one diagnosis after the first diagnosis visit was higher in the PPA than in the AD group. Diagnoses registered after a PPA diagnosis were most often PPA (72.7%), then AD then behavioural variant of frontotemporal neurocognitive disorder then mixed neurocognitive disorder. And after AD diagnosis, it was most frequent AD diagnosis (90.7%) then mixed neurocognitive disorder (Table 2).

### Evolution in treatment

Patients with at least one BNA record before and after the diagnosis of interest and for whom the data about pharmacological (*N*=1622) and non-pharmacological treatment (*N*=1605) were registered were selected. The number of patients under pharmacological treatment was significantly higher after, than before the diagnosis of PPA, and this is true for the different psychotropic drugs and for anti-dementia treatments. After diagnosis, the treatment that was mainly added in the PPA group was antidepressants (for 20,6% of the patients). More patients received non-pharmacological treatments after a PPA compared to before the diagnosis, with the most common intervention being speech-language therapy (see Table 3). The delay between diagnosis and starting speech-language therapy was 6.9 (± 6.3) months.

**Table 3:** Treatments before and after first consultation with diagnosis of interest

	Before first consultation with diagnosis of interest		After first consultation with diagnosis of interest		
	*n*	(%)	*n*	(%)	*p*-value
**PPA patients**					
**Pharmacological treatments**					
Antidepressant	330	(20.4)	629	(38.8)	<.001
Anxiolytic	130	(8.0)	237	(14.6)	<.001
Hypnotic	64	(4.0)	94	(5.8)	<.001
Neuroleptic	29	(1.8)	116	(7.2)	<.001
NMDA antagonist	77	(4.8)	306	(18.9)	<.001
ChEI	144	(8.9)	426	(26.3)	<.001
**Non-pharmacological treatments**					
Day hospital	24	(1.5)	158	(9.8)	<.001
Speech and language therapist (SLT)	365	(22.7)	1060	(66.0)	<.001
Psychologist	66	(4.1)	130	(8.1)	<.001
Kinesiologist	35	(2.2)	169	(10.5)	<.001
Occupational therapist	17	(1.1)	50	(3.1)	<.001
Stakeholder group	24	(1.5)	84	(5.2)	<.001
**AD patients**					
**Pharmacological treatments**					
Antidepressant	10,261	(24.1)	16,528	(38.8)	<.001
Anxiolytic	5226	(12.3)	8626	(20.3)	<.001
Hypnotic	2375	(5.6)	3380	(7.9)	<.001
Neuroleptic	1359	(3.2)	3943	(9.3)	<.001
NMDA antagonist	1801	(4.2)	11,670	(27.4)	<.001
ChEI	5967	(14.0)	24,927	(58.6)	<.001
**Non-pharmacological treatments**					
Day hospital	628	(1.5)	5272	(12.7)	<.001
Speech and language therapist (SLT)	2,785	(6.7)	8807	(21.2)	<.001
Psychologist	1214	(2.9)	2377	(5.7)	<.001
Kinesiologist	1562	(3.8)	4311	(10.4)	<.001
Occupational therapist	658	(1.6)	1546	(3.7)	<.001
Stakeholder group	941	(2.3)	3038	(7.3)	<.001

*McNemar test

In the AD group too, the number of patients under pharmacological treatment (*N*=42,571) was significantly higher after, than before the diagnosis (Table 3), and this is also true for the different psychotropic drugs and for anti-dementia treatments. After diagnosis, the treatment that was mainly added was cholinesterase inhibitors (for 46.4%of the AD patients). As for the PPA group, more patients received non-pharmacological treatments after receiving an AD diagnosis compared to before the diagnosis. The delay between diagnosis and starting speech-language therapy was 9.0 (± 9.6) months.

## Discussion

The present study, based on data gathered in the BNA, showed an incidence of PPA of 1.14/100,000 person-year, which is consistent with that was previously reported in FTLD and can be estimated in PPA [5, 12, 13].

Median age at first diagnosis was significatively lower in the PPA than in the AD group, which is in agreement with other studies that have shown that symptoms onset happened at a younger age in PPA than in AD [16]. However, the onset of PPA is known to occur before the age of 65 [17] and the disease is described as a progressive language deficit occurring between 45 and 70 years old [14]. So, our results are not aligned to those of previous studied cohort and indicates possible bias of recruiting older people in memory centres, because they are mostly known as a diagnostic facility for memory and not language disorders.

The other possible explanation would be the proportion of different PPAs in the database and the lack of literature on the age of onset of an lvPPA.

Sex ratio was more balanced in the PPA than in the AD group, with 55.7% of females. Unless a majority of women was described as usual, sex ratio varies from one cohort to another in AD, in PPA group our results are in agreement with the literature [3].

The educational level was higher in the PPA than in the AD group, which is more than described in other studies [3] but in agreement with the fact of early onset dementia patients are more educated than patients with late onset dementia [18]. So, compared to other studies, we can suppose that PPA will be able to cope better with greater brain damage than AD [19].

The mean MMSE score (in 2-point difference) at first diagnosis was not statistically different in the PPA than in the AD group, which is in agreement with the elements found in the literature [20]. MMSE’s item scores could be useful to discriminate PPA patients and AD [21, 22] but it was not possible to test this in this study [21, 22].

About evolution of PPA diagnosis, the most frequently, the diagnosis stays PPA (72.7%), including temporal variant of FTLD, then AD then behavioural variant of FTLD. These results are in agreement with literature values [3].

Regarding the initially diagnosed PPA patients, the natural evolution to a FTD in temporal or frontal variant diagnosis is concordant as the language impairment will be accompanied by a more global cognitive deficit. In cognitively impaired patients, the appearance of a global cognitive deficit or behavioural symptoms announces a future onset of dementia in the six following years of evolution, mostly with a frontal phenotype (75%) [23].

The second evolution observed in our study is AD with 13.8%. These results are in agreement with the fact of due to similar neuropathology and the clinical phenotype presented after the aphasic state, the logopenic subtype of PPA is considered as an atypical form of AD [24].

Other evolutive profile are also reported in our study like Lewy body dementia (0.5%), corticobasal degeneration (CBD) (0.5%) and progressive supranuclear palsy (0.5%) in agreement with literature [25, 26].

Indeed, some authors consider PPA-plus syndromes when aphasia is still not the only major deficit. They report that disinhibition by familiarity, blunted judgement and difficulty in problem solving result in a personality change. Personality change and asymmetric extrapyramidal deficit occur frequently considering the PPA anatomical dysfunction evolution, which is supposed to be close to behavioural variant of FTD or CBD [27].

Also, initial language deficits are reported in one third of corticobasal dementia.

However, in our study, some results are surprising, as the percentage of evolution in subjective complaint which is not a mode of evolution of PPAs and can bring into question on a possible diagnostic error at the beginning.

Our secondary objectives were to determine if diagnosis of PPA is more difficult to establish and more delayed than AD and what are the related prescribed treatment over 7 years of follow-up.

For patients diagnosed of PPA at the end of the follow-up, the first diagnosis was 12.7% Alzheimer’s dementia, 7.1% subjective memory complaint and 4.4% non-amnestic mild cognitive impairment. Subjective memory complaint in the cohort is corresponding to the first consultation of the patient that could, at this time, stay at a subjective cognitive impairment report. We know that PPA patients can complain of memory loss and may perform poorly in standard tests of memory [28]. However, percentage of patients initially diagnosed with AD or with a pending diagnosis (49.6%) seem to support the hypothesis that diagnosis of PPA is more difficult and more delayed than AD.

Other results seem to support this hypothesis, like:

- The delay between the first consultation for cognitive troubles and the first diagnosis that is significantly longer than in the AD group.
- The number of different diagnoses before the diagnosis of interest that is significantly higher in the PPA group than in the AD group.
- The percentage of patients with more than one diagnosis that was higher in the PPA group than in the AD group.

To summarize, despite the evolution of the diagnostic criteria of primary progressive aphasia over time [14, 15], their diagnostic still does not seem optimal.

Our study shows also that PPA diagnosis is particularly important because it modifies pharmacological and nonpharmacological interventions.

Indeed, the number of patients under pharmacological treatment increases of more than 50% after the diagnosis of PPA. Also, non-pharmacological treatments increase after the diagnosis and especially speech therapy (22.7% before and 66.0% after).

In the field of pharmacological interventions, to date, no medications have been shown to improve or stabilize cognitive deficits in patients with PPA [29]. Indeed, clinical trials on patients with FTD and controlled trials on PPA patients with bromocriptine [30] and memantine [31] have not demonstrated any efficiency. Studies on limited patient samples with galantamine, rivastigmine and selegiline have shown no results [32]. Also, although some patients with PPA, particularly with a logopenic subtype, may suffer from atypical AD, cholinesterase inhibitors have shown no results. No effect is expected as cholinergic deficit has been specifically identified in AD’s physiopathology. Worsening of behavioural variant of FTD is also suspected under anticholinerterasic treatment. So on, actual recommendation is not favourable for anticholinesterase inhibitors’ use in FTD [33].

However, benefits have been found on behaviour but not on cognition with the cholinesterase inhibitor rivastigmine and the MAO-B inhibitor selegiline [34].

The use of antidepressants, such as trazadone, is known to be effective on the behavioural symptoms but no impact on cognition is reported in FTD [35]. Antipsychotics with careful and limited use could improve behavioural symptoms but their side effect on wake and cognition limits their use. It is suspected that high-frequency repetitive transcranial magnetic stimulation (hf-rTMS) applied to the left prefrontal cortex produces improvement on language test that seems to last; other magnetic stimulations are suspected to maintain some language capacities [36].

In summary, although no drugs have shown an effectiveness on PPA, but, on behavioural disorders, some drugs have been deleterious, and others had positive effects. It thus appears essential to establish a diagnosis of PPA to set up an adapted medical treatment.

A key intervention on the PPA population is speech and language therapy (SLT): a specific form of cognitive intervention that evaluates communication skills and designs a personalized intervention plan to improve communication abilities. This type of intervention has been shown to be effective and advised to be implemented in a more systematic way [37].

Also, in addition to the take care of language disorders, speech therapy is particularly important for swallowing disorders, which represent vital risks. Indeed, all variants showed swallowing difficulties and they were more frequent in PPA-S [38].

Finally, the put of diagnosis is essential whether it is for more adapted pharmacological or non-pharmacological interventions.

Logopenic variant of PPA (lv-PPA) is a neurodegenerative syndrome frequently associated with biomarkers of AD. Lv-PPA patients display characteristic linguistic deficits, a pattern of brain atrophy, and possibly genetic susceptibility, which warrant considering this variant as a discrete AD endophenotype [39]. Also, recent diagnostic criteria include lvPPA as an atypical early onset variant of AD because sporadic lvPPA clinical syndrome is both associated with AD biomarkers and AD pathological changes in about 85–90% of cases [8, 40–42]. For these reasons, patients with PPAs are often included in studies on AD.

However, it has been shown that the classification of lvPPA does not successfully differentiate PPA due to AD from PPA due to other pathologies [43]. Furthermore, several underlying neurodegenerative etiologies have been reported in a few lvPPA cases, which can be linked to Lewy body disorder [44] and coexisting disorders or to biomarkers discordant with the clinical syndrome, especially in older individuals [42, 45].

Understanding in vivo pathological prediction is crucial in neurodegenerative diseases because therapeutic pharmacological strategies are, or soon will be, directed towards decreasing or clearing toxic molecules, such as amyloid, tau or TDP. This study highlights importance to improve early diagnosis of PPA to better understand links with AD.

Finally, studies have demonstrated that toxic proteins including amyloid, tau and TDP43 spread transneuronally through connected networks in a prion-like manner [46, 47]. In PPA neuroimaging support these findings by showing network-specific damage. The studies comparing PPAs and ADs seem even more of interest because they permit to investigate the intricate relationship between protein deposition patterns and network susceptibility in neurodegenerative diseases. In summary, the comparison of clinical characteristics between PPA and AD patients could provide a better understanding of the reasons for network susceptibility generating clinical disorders increased on the language or memory.

### Limitations of the present study

Despite BNA represents a valuable epidemiologic tool because it grants access to many patients with dementia and permits follow-up studies, several limitations should be noted.

First, data are entered into the BNA by different physicians and even though they all follow standard criteria for diagnosis, there is no external validation that those criteria were met. Also, criteria for diagnosis of PPA have be modified since 2010. Second, even though the BNA includes the great majority of individuals with PPA and associated disorders who are referred to specialized centres (French memory units), individuals included in the BNA are not fully representative of the total French population with PPA. Indeed, one part of the population with PPA is under *general practitioner* (GP) supervision only (GPs do not currently have access to the BNA), and another part of the population is referred to specialists (geriatricians, neurologists, psychiatrists) who are not using the BNA database. Finally, the data reported in the BNA do not include the information on the PPA variants and thus do not allow to perform separate analyses for different patients’ groups.

## Conclusion

This study provided data on the clinical characteristics and the evolution of PPAs over a very large cohort.

It has also highlighted:

1. The current difficulty of making a diagnosis of PPA because of the varied symptomatology of the three variants and the underlying pathology (FTD or AD).
2. The use of poorly adapted diagnostic and severity assessment tools due to verbal instructions and proposed language tasks.
3. The importance of early diagnosis between PPA and AD due to differences in therapeutic approaches.

The perspectives of this study are to develop tools of diagnosis and severity assessment more adapted by including early markers of the pathology (graphic markers and vocal markers).

## Supplementary Information

**Additional file 1: Table S1.** Comparison of descriptive characteristics of the AD vs PPA groups (Bayesian analyses). **Table S2.** Number of different diagnoses before and after first consultation.

## Data Availability

The data reported are part of an ongoing registration programme. Deidentified participant data are not available for legal and ethical reasons. Anonymized data will be made available for research purposes, upon request and specific approval of the database advisory board and ethical committee.

## References

[1] Ratnavalli E, Brayne C, Dawson K, Hodges JR (2002). The prevalence of frontotemporal dementia. Neurology..

[2] Mesulam M-M, Rogalski EJ, Wieneke C, Hurley RS, Geula C, Bigio EH (2014). Primary progressive aphasia and the evolving neurology of the language network. Nat Rev Neurol.

[3] Le Rhun E, Richard F, Pasquier F (2005). Natural history of primary progressive aphasia. Neurology..

[4] Dubois B, Padovani A, Scheltens P, Rossi A, Dell’Agnello G (2015). Timely diagnosis for Alzheimer’s disease: a literature review on benefits and challenges. Saykin A, editor. J Alzheimer’s Dis.

[5] Kertesz A, Morlog D, Light M, Blair M, Davidson W, Jesso S (2008). Galantamine in frontotemporal dementia and primary progressive aphasia. Dement Geriatr Cogn Disord.

[6] Grossman M (2010). Primary progressive aphasia: clinicopathological correlations. Nat Rev Neurol.

[7] Dubois B, Feldman HH, Jacova C, Cummings JL, Dekosky ST, Barberger-Gateau P (2010). Revising the definition of Alzheimer’s disease: a new lexicon. Lancet Neurol.

[8] Spinelli EG, Mandelli ML, Miller ZA, Santos-Santos MA, Wilson SM, Agosta F (2017). Typical and atypical pathology in primary progressive aphasia variants: pathology in PPA variants. Ann Neurol.

[9] Mesulam M (2013). Primary progressive aphasia: a dementia of the language network. Dement Neuropsychol.

[10] Weintraub S, Rubin NP, Mesulam MM (1990). Primary progressive aphasia. Longitudinal course, neuropsychological profile, and language features. Arch Neurol.

[11] Spinney L (2008). Alzheimer’s disease funding and the French health system. Lancet Neurol.

[12] Folstein MF, Folstein SE, McHugh PR (1975). “Mini-mental state”. A practical method for grading the cognitive state of patients for the clinician. J Psychiatr Res.

[13] Le Duff F, Develay AE, Quetel J, Lafay P, Schück S, The participating centers (2012). The 2008–2012 French Alzheimer plan: description of the national Alzheimer information system. J Alzheimers Dis.

[14] Mesulam MM (2001). Primary progressive aphasia. Ann Neurol.

[15] Gorno-Tempini ML, Hillis AE, Weintraub S, Kertesz A, Mendez M, Cappa SF (2011). Classification of primary progressive aphasia and its variants. Neurology..

[16] Hommet C, Mondon K, Perrier D, Rimbaux S, Autret A, Constans T (2008). L’aphasie progressive primaire : un cadre à part dans les pathologies neurodégénératives. La Revue de Médecine Interne.

[17] Mesulam M-M, Wieneke C, Thompson C, Rogalski E, Weintraub S (2012). Quantitative classification of primary progressive aphasia at early and mild impairment stages. Brain..

[18] Maiovis P, Ioannidis P, Konstantinopoulou E, Karacostas D (2015). Early onset degenerative dementias: demographic characteristics and etiologic classification in a tertiary referral center. Acta Neurol Belg.

[19] Maiovis P, Ioannidis P, Gerasimou G, Gotzamani- Psarrakou A, Karacostas D (2018). Cognitive reserve hypothesis in frontotemporal dementia: evidence from a brain SPECT study in a series of Greek frontotemporal dementia patients. Neurodegener Dis.

[20] Vigliecca NS, Peñalva MC, Molina SC, Voos JA, Vigliecca MR (2012). Is the Folstein’s mini-mental test an aphasia test?. Appl Neuropsychol Adult.

[21] Macoir J, Fossard M, Lefebvre L, Monetta L, Renard A, Tran TM (2017). Detection test for language impairments in adults and the aged—a new screening test for language impairment associated with neurodegenerative diseases: validation and normative data. Am J Alzheimers Dis Other Dement.

[22] Flanagan EC, Tu S, Ahmed S, Hodges JR, Hornberger M (2014). Memory and orientation in the logopenic and nonfluent subtypes of primary progressive aphasia. JAD..

[23] Signoret J-L, Allard M, Benoit N, Bolgert F (1989). Evaluation des troubles de mémoire et des désordres cognitifs associés: B.E.C. 96.

[24] McKhann GM, Knopman DS, Chertkow H, Hyman BT, Jack CR, Kawas CH (2011). The diagnosis of dementia due to Alzheimer’s disease: recommendations from the National Institute on Aging-Alzheimer’s association workgroups on diagnostic guidelines for Alzheimer’s disease. Alzheimers Dement.

[25] Kertesz A, Davidson W, Mccabe P, Takagi K, Munoz D (2003). Primary progressive aphasia: diagnosis, varieties, evolution. J Int Neuropsychol Soc.

[26] Knibb JA, Xuereb JH, Patterson K, Hodges JR (2006). Clinical and pathological characterization of progressive aphasia. Ann Neurol.

[27] Mesulam M-M (2003). Primary progressive aphasia — a language-based dementia. N Engl J Med.

[28] Weintraub S, Rogalski E, Shaw E, Sawlani S, Rademaker A, Wieneke C (2013). Verbal and nonverbal memory in primary progressive aphasia: the three words-three shapes test. Behav Neurol.

[29] Shigaeff N, Zanetti M, Tierno S d A, Tommaso ABGD, Marques TC, Franco FG (2017). An interdisciplinary approach aiding the diagnosis of primary progressive aphasia: a case report. Dement Neuropsychologia.

[30] Reed DA, Johnson NA, Thompson C, Weintraub S, Mesulam M-M (2004). A clinical trial of bromocriptine for treatment of primary progressive aphasia. Ann Neurol.

[31] Johnson NA, Rademaker A, Weintraub S, Gitelman D, Wienecke C, Mesulam M (2010). Pilot trial of memantine in primary progressive aphasia. Alzheimer Dis Assoc Disord.

[32] Birks J, Flicker L. Selegiline for Alzheimer’s disease. Cochrane Database Syst Rev. 2003;(1). SN 1465-1858. Art. No.: CD000442. 10.1002/14651858.CD000442 12535396

[33] Kerchner GA, Tartaglia MC, Boxer AL (2011). Abhorring the vacuum: use of Alzheimer’s disease medications in frontotemporal dementia. Expert Rev Neurother.

[34] Moretti R, Torre P, Antonello RM, Cattaruzza T, Cazzato G, Bava A (2004). Rivastigmine in frontotemporal dementia: an open-label study. Drugs Aging.

[35] Lebert F, Stekke W, Hasenbroekx C, Pasquier F (2004). Frontotemporal dementia: a randomised, controlled trial with trazodone. Dement Geriatr Cogn Disord.

[36] Köhler TS, Choy JT, Fazili AA, Koenig JF, Brannigan RE (2012). A critical analysis of the reported association between vasectomy and frontotemporal dementia. Asian J Androl.

[37] Kiousi V, Arnaoutoglou M, Printza A (2019). Speech and language intervention for language impairment in patients in the FTD-ALS spectrum. Hell J Nucl Med.

[38] Marin S, Bertolucci PHF, Marin LF, de Oliveira FF, Wajman JR, Bahia VS (2016). Swallowing in primary progressive aphasia. NeuroRehabilitation..

[39] Leyton CE, Hodges JR (2013). Towards a clearer sefinition of logopenic progressive aphasia. Curr Neurol Neurosci Rep.

[40] Dubois B, Feldman HH, Jacova C, Hampel H, Molinuevo JL, Blennow K (2014). Advancing research diagnostic criteria for Alzheimer’s disease: the IWG-2 criteria. Lancet Neurol.

[41] Wilson SM, Dronkers NF, Ogar JM, Jang J, Growdon ME, Agosta F (2010). Neural correlates of syntactic processing in the nonfluent variant of primary progressive aphasia. J Neurosci.

[42] Bergeron D, Gorno-Tempini ML, Rabinovici GD, Santos-Santos MA, Seeley W, Miller BL (2018). Prevalence of amyloid-β pathology in distinct variants of primary progressive aphasia. Ann Neurol.

[43] Harris JM, Gall C, Thompson JC, Richardson AMT, Neary D, du Plessis D (2013). Classification and pathology of primary progressive aphasia. Neurology..

[44] Giannini L, Irwin D, McMillan C, Ash S, Wolk D, Isenberg A (2016). Clinicopathological correlations of AD neuropathology in the logopenic variant of primary progressive aphasia (S39.002). Neurology..

[45] Santos-Santos MA, Rabinovici GD, Iaccarino L, Ayakta N, Tammewar G, Lobach I (2018). Rates of amyloid imaging positivity in patients with primary progressive aphasia. JAMA Neurol.

[46] Smethurst P, Newcombe J, Troakes C, Simone R, Chen Y-R, Patani R (2016). In vitro prion-like behaviour of TDP-43 in ALS. Neurobiol Dis.

[47] Ruiz-Riquelme A, Lau HHC, Stuart E, Goczi AN, Wang Z, Schmitt-Ulms G (2018). Prion-like propagation of β-amyloid aggregates in the absence of APP overexpression. Acta Neuropathol Commun.

