# Supplementary material for: The course of primary progressive aphasia diagnosis: a cross-sectional study
Source: Alzheimers Res Ther. 2022 May 10;14:64. doi: 10.1186/s13195-022-01007-6 (PMC9092839; doi:10.1186/s13195-022-01007-6)
Supplement: Supplementary file 1 — Additional file 1: Table S1. Comparison of descriptive characteristics of the AD vs PPA groups (Bayesian analyses). Table S2. Number of different diagnoses before and after first consultation. [file 13195_2022_1007_MOESM1_ESM.docx]

Additional Table 1: Comparison of descriptive characteristics of the AD vs PPA groups (Bayesian analyses)

|  | AD vs PPA |
| --- | --- |
| **Probability to get a difference of 5%** |  |
| Sexe | 1 |
| Community-living | 1 |
| Location of the patient | 1 |
| Center: Memory clinic vs Regional specialized memory clinic/Private practice neurologist | 1 |
| Center: Regional specialized memory clinic vs Memory clinic/Private practice neurologist | 1 |
| Center: Private practice neurologist vs Regional specialized memory clinic/Memory clinic | <0,001 |
| Initially referred by: General practitioner vs all others | 1 |
| Initially referred by: Neurologist vs all others | 1 |
| Initially referred by: Other specialists vs all others | <0,001 |
| Initially referred by: Direct vs all others | <0,001 |
| Education: No education vs all others | <0,001 |
| Education: Primary vs all others | 1 |
| Education: Secondary first cycle vs all others | 0,039 |
| Education: Secondary second cycle vs all others | 0,999 |
| Education: Superior vs all others | 1 |
| **Probability to get a difference of** |  |
| 2 years for the age | 1 |
| 2 points for MMSE | <0,001 |
| 1 points for MMSE | 1 |

Abbreviations: AD=Alzheimer's Disease, PPA=Primary Progressive Aphasia, MMSE=Mini Mental State Examination.

The probability corresponds to the numbers of iterations where the difference of 5% for qualitative variables and 2 points for quantitative variables were observed among all completed iterations. A probability = 1 means all iterations show the difference whereas a probability = 0 means no iteration shows the difference

Additional Table 2: Number of different diagnoses before and after first consultation.

|  | PPA (n=5,186) | | AD (n=162,005) | |  |
| --- | --- | --- | --- | --- | --- |
|  | Mean | [SD] | Mean | [SD] | p-value |
| **Period between first consultation and first diagnosis (years)** | 0.7 | [1.4] | 0.6 | [1.4] | <.001 |
|  | n | (%) | n | (%) | p-value |
| **Number of different diagnoses BEFORE first consultation with diagnosis of interest** |  |  |  |  | <.001 |
| 0 | 2,892 | (55.8) | 98,775 | (61.0) |  |
| 1 | 1,890 | (36.4) | 54,709 | (33.8) |  |
| 2 | 338 | (6.5) | 7,536 | (4.7) |  |
| 3 | 54 | (1.0) | 880 | (0.5) |  |
| 4 | 10 | (0.2) | 95 | (0.1) |  |
| 5 | 2 | (0.0) | 10 | (0.0) |  |
| **Number of different diagnoses AFTER first consultation with the diagnosis of interest** |  |  |  |  | <.001 |
| 0 | 3,971 | (76.6) | 147,235 | (90.9) |  |
| 1 | 992 | (19.1) | 13,234 | (8.2) |  |
| 2 | 186 | (3.6) | 1,344 | (0.8) |  |
| 3 | 33 | (0.6) | 164 | (0.1) |  |
| 4 | 2 | (0.0) | 27 | (0.0) |  |
| 5 | 2 | (0.0) | 1 | (0.0) |  |
